# 

**Published:** 1893-03

**Authors:** 


### INDEX TO VOLUME X.

ORIGINAL COMMUNICATIONS—

Abortion in an Hour (Harris) . . . . . . . . . . . . . . . . . . . . 211
Abuse of Astringents in Treatment of Catarrh (Shields) . . . . . . . . 86
Animal Extracts (Hammond) . . . . . . . . . . . . . . . . . . . . 5
Ascites in Connection with Gynecology (Gusserow) . . . . . . . . . . 69
Board of Medical Examiners (Grandy) . . . . . . . . . . . . . . . . 129
Breast, Carcinomatous Tumors of, Operative Procedures for (Gaston) . . 579
Cases (Surgical) from Practice (Hurt) . . . . . . . . . . . . . . . 76
Cholera (Hart) . . . . . . . . . . . . . . . . . . . . . . . . 321
Cocaine in Surgery (Auber) . . . . . . . . . . . . . . . . . . . . 88
Condylomata, Syphilitic, of the Auditory Canal (Roy) . . . . . . . . . 461
Cystitis and Urethritis, a Case of Chronic, with Ulcers (Hurt) . . . . 715
Enterectomy—End to End Anastomosis—Cholecyst-Enterostomy with Murphy Button (Rogers).. . . . . . . . . . . . . . . . . . . . . 648
Epithelioma, Obstructive, of the Ileo-cecal Valve, Enterectomy for, Secondary Anastomosis by Abbe's Long Incision (Barton) . . . . . . . . 456
Extra-Uterine Pregnancy and Pyosalpinx (Kime) . . . . . . . . . . . 148
Fever, Hemorrhagic (Bush) . . . . . . . . . . . . . . . . . . . . 396
Fever, Typhoid (Conway) . . . . . . . . . . . . . . . . . . . . . 521
Fever, Typhoid, The Diazo-Reaction in (Friedenwald) . . . . . . . . . 655
Four Women Who Refused Oöphorectomy (McHatton) . . . . . . . . . 273
Generative Organs, Female, Chancroids of (Davis) . . . . . . . . . . 587
Goitre, Exophthalmic, Abstracts of Notes on the Treatment of (Taylor) 526
Gonorrhea (McRae) . . . . . . . . . . . . . . . . . . . . . . . . 200
Hemorrhage, Spinal, from Traumatic Influence, a few Clinical Notes on the Usual Situation of (Manley) . . . . . . . . . . . . . . . . . . 449
Hepatic Abscess (Bloch) . . . . . . . . . . . . . . . . . . . . . . 337
Kidney, Movable, the Diagnosis and Treatment of (Holmes) . . . . . . 641
Medicine, the Practice of in Georgia (Blain) . . . . . . . . . . . . . 385
Urethrotomy, External *versus* Internal (Dugan) . . . . . . . . . . . . 709
Umbilical Cord, the Best Treatment of (Hurt) . . . . . . . . . . . . 594
New Facts about Old Remedy, Tincture of Iron (Starr) . . . . . . . . . 14
Optic Nerve, Choroid and, Persistent Papillary Membrane Associated with Atrophy of (Phillips) . . . . . . . . . . . . . . . . . . . . 652
Pharynx and Larynx, Foreign Bodies in (Hobbs and Brown). . . . . . 705
Physician's Life, A, the Prose and Poetry of (Webb). . . . . . . . . . 513
Psoriasis, The Local Treatment of (Longfellow). . . . . . . . . . . . 398
Puerperal Septicemia (Darby) . . . . . . . . . . . . . . . . . . . 193
Puerperium, The Classification of Infection during, with Notes on Treatment (Kime). . . . . . . . . . . . . . . . . . . . . . . . 528
Pyosalpinx Relieved by Drainage through Vagina (Davis) . . . . . . 83

ORIGINAL COMMUNICATIONS—CONTINUED—

Relations of Operative Gynecology to Insanity (McFarland) . . . . . 269
Science in Medicine and Surgery (Gaston) . . . . . . . . . . . . . . 141
Spine, Injuries of the (Smith) . . . . . . . . . . . . . . . . . . . . 722
State and Municipal Hygiene (Avary). . . . . . . . . . . . . . . . 261
Strychnine, An Approximate Estimate of the Energy Contained in (Barr) 406
Surgical Clinic at Rush Medical College (Hamilton). . . . . . . . . . 65
Surgical Clinic at Harlem Hospital (Manley) . . . . . . . . . . . 1, 257, 577
Syphilis by Conception, A Case of (Chambrelent). . . . . . . . . . . 717
Uses of Water as Therapeutic Agent (Stockard). . . . . . . . . . . 206
Uterine Fibroma (Murfree) . . . . . . . . . . . . . . . . . . . . . 18
Uterus, Ante- and Retro-positions of. Their Pathology, Symptomatology and Treatment (Stewart). . . . . . . . . . . . . . . . . . . . 327, 390
What the Practitioner Should Know about Diseases of the Eye (Smith) . 340

EDITORIAL—

American *vs.* Foreign Pharmaceutical Articles . . . . . . . . . . . . 169
Anesthesia, The Discovery of Modern Surgical . . . . . . . . . . . 545
Association, Southern Surgical and Gynecological . . . . . . . . . . 617
Atlanta Polyclinic . . . . . . . . . . . . . . . . . . . . . . . . . 99
Bill, The Legislature and the . . . . . . . . . . . . . . . . . . . 619
Bill, The Defeat of the . . . . . . . . . . . . . . . . . . . . . . 673
Board of Examiners, The Legislature and a . . . . . . . . . . . . . 547
Buccaneer, The Medical . . . . . . . . . . . . . . . . . . . . . . 736
Chloroform Commission, Report of the Third Hyderabad . . . . . . . 615
Comments from the West . . . . . . . . . . . . . . . . . . . . . . 359
Congress, The Pan-American Medical . . . . . . . . . . . . . . . . 480
Doctor, The, as a Business Man . . . . . . . . . . . . . . . . . . 674
Dermatology . . . . . . . . . . . . . . . . . . . . . . . . . . . . 675
Eclectic Review . . . . . . . . . . . . . . . . . . . . . . . . . . 35
Encouraged . . . . . . . . . . . . . . . . . . . . . . . . . . . . 235
Examining Board, The, and the Public Press . . . . . . . . . . . . 420
Florence Crittenton Home, The . . . . . . . . . . . . . . . . . . . 548
Fever, The, at Brunswick . . . . . . . . . . . . . . . . . . . . . 479
Fever . . . . . . . . . . . . . . . . . . . . . . . . . . . . . . . 417
From a Sister State . . . . . . . . . . . . . . . . . . . . . . . . 737
Journal and the Colleges . . . . . . . . . . . . . . . . . . . . . 233
Library, Medical, for the State . . . . . . . . . . . . . . . . . . 481
Lister on Antisepsis . . . . . . . . . . . . . . . . . . . . . . . 291
Medical Journalism . . . . . . . . . . . . . . . . . . . . . . . . 356
Medical College Commencement . . . . . . . . . . . . . . . . . . . 38
Meeting of the American Medical Association . . . . . . . . . . . . 290
Meeting of the Association . . . . . . . . . . . . . . . . . . . . . 165
Pan-American Medical Congress . . . . . . . . . . . . . . . . . . . 37
Professional Education in the United States . . . . . . . . . . . . 234
Quack Doctors' Methods . . . . . . . . . . . . . . . . . . . . . . 100
State Medical Association . . . . . . . . . . . . . . . . . . . . . 98
United Confederate Veterans . . . . . . . . . . . . . . . . . . . . 358

CORRESPONDENCE.—

A Monument to the Memory of the Late Dr. Willis F. Westmoreland . . 416
An Interesting Case . . . . . . . . . . . . . . . . . . . . . . . . . 671
Case of Cretinism . . . . . . . . . . . . . . . . . . . . . . . . . . 31
Case for Diagnosis and Treatment . . . . . . . . . . . . . . . . . . 354
Obstinate Case of Constipation . . . . . . . . . . . . . . . . . . . . 288
New York Letters . . . . . . . . . . . . 26, 228, 282, 350, 539, 609, 666
The Willis F. Westmoreland Memorial . . . . . . . . . . . . . . . . . 544
Two Interesting Cases . . . . . . . . . . . . . . . . . . . . . . . . 96
Valvular Insufficiency and Anasarca . . . . . . . . . . . . . . . . . 287

SOCIETY REPORTS—

Academy of Surgery, Philadelphia . . . . . . . . . . . . . . . . . 402
Atlanta Obstetrical Society, Transactions of . . . . . . . . . . . . 409 469,
Atlanta Society of Medicine . . . . . . . . . . . . . . . . . . . . . 214, 724
Clinical Society of Maryland. . . . . . . . . . . . . . . . . . . . . 20
Georgia State Medical Association . . . . . . . . . . . . . . . . . . 152
Macon Medical Society . . . . . . . . . . . . . . . . . . . . . . . . 729
Orleans Parish Medical Society . . . . . . . . . . . . . . . . . . . 343, 658
Philadelphia Academy of Surgery . . . . . . . . . . . . . . . . . . . 276
Richmond Academy of Medicine and Surgery. . . . . . . . . . . . . . . 93
Southern Surgical and Gynecological Association . . . . . . . . . . . 595

SELECTIONS AND ABSTRACTS—

Eye, Ear, Nose and Throat . . . . . . . . . 120, 179, 239, 310, 361, 441, 505
General Medicine and Therapeutics . . . . . . 40, 113, 173, 237, 302, 376, 438, 499, 560, 621, 682
Obstetrics and Gynecology . . . . . . . 50, 55, 107, 170, 246, 306, 364, 429, 487, 687, 743
Practical Chemistry and Pharmacy . . . . . . . . . . . . . . 568, 636, 761
Skin Diseases and Syphilis . . . . . . 117, 177, 242, 294, 369, 434, 483, 549, 627, 677, 739
Surgery . . . . . . . . 46, 103, 248, 297, 372, 422, 494, 555, 631, 696, 754

MEDICAL ITEMS . . . . 59, 122, 183, 251, 379, 444, 509, 569, 637, 702, 764

BOOK REVIEWS . . . . . . 62, 124, 186, 253, 381, 446, 572, 639, 676, 703

### CONTRIBUTORS TO VOLUME X.

---

| | |
|---|---|
| Hobbs, Arthur G., M. D | Atlanta |
| Brown, George, M. D | Atlanta |
| Dugan, W. C., M. D | Louisville, Ky |
| Hurt, C. D., M. D | Atlanta |
| Chambrelent, Professor, (Translation) | Bordeaux, France |
| Smith, J. Y. H | Rochelle, Ga |
| Holmes, J. B. S | Atlanta |
| Rodgers, W. B | Memphis, Tenn |
| Phillips, S. Latimer | Savannah, Ga |
| Freidenwald, Julius | Baltimore |
| Manley, Thomas H | New York |
| Russell, Wm. L | New York |
| Gaston, J. McF., Sr | Atlanta |
| Davis, E. C | Atlanta |
| Webb, Charles S | Atlanta |
| Conway, Wm. B | Athens, Ga |
| Taylor, J. Madison | Philadelphia |
| Kime, R. R | Atlanta |
| Barton, James M | Philadelphia |
| Roy, Dunbar | Atlanta |
| Barr, A. D | Calamine, Ark |
| Blain, Arthur C | Macon, Ga |
| Stewart, W. W | Columbus, Ga |
| Bush, O. B | Arlington, Ga |
| Longfellow, R. C | Cincinnati, O |
| Hamilton, John B | Chicago, Ill |
| Gusserow, A | Berlin, Germany |
| Davis, J. S | Montevallo, Ala |
| Shields, Charles M | Richmond, Va |
| Darby, J. I | Americus, Ga |
| McRae, F. W | Atlanta, Ga |
| Stockard, C. C | Atlanta |
| Harris, Charles H | Cedartown, Ga |
| Manley, Thomas H | New York, N. Y |
| Avary, J. C | Atlanta, Ga |
| McFarland, A. H | Jacksonville, Ill |
| McHatton, H | Macon, Ga |
| Hammond, W. A | Washington, D. C |
| Starr, E. F | Nacoochee, Ga |
| Murfree, J. B | Murfreesboro, Tenn |
| Grandy, L. B | Atlanta, Ga |
| Hart, Ernest | London, England |
| Bloch, A. J | New Orleans, La |
| Smith, F. T | Chattanooga, Tenn |

